# Searching chromosome mosaicisms in 45,X Turner syndrome: how relevant is it?

**DOI:** 10.20945/2359-3997000000403

**Published:** 2021-11-11

**Authors:** Jéssica Silva Soares, Renata Maria Rabello da Silva Lago, Maria Betânia Pereira Toralles, Laís Ribeiro Mota, Esmeralda Santos Alves, Acácia Fernandes Lacerda de Carvalho

**Affiliations:** 1 Universidade Federal da Bahia Instituto de Biologia Laboratório de Genética Humana e Mutagênese Salvador BA Brasil Laboratório de Genética Humana e Mutagênese, Instituto de Biologia, Universidade Federal da Bahia, Salvador, BA, Brasil; 2 Universidade Federal da Bahia Hospital Universitário Edgard Santos Departamento de Genética Médica Salvador BA Brasil Departamento de Genética Médica, Hospital Universitário Edgard Santos, Universidade Federal da Bahia, Salvador, BA, Brasil

**Keywords:** Turner syndrome, PCR, FISH technique, mosaicism, monosomy X

## Abstract

**Objective:**

To investigate the presence of chromosome mosaicism, especially for the presence of Y derived material in 45,X women with Turner syndrome (TS).

**Materials and methods:**

FISH and PCR were performed for the presence of chromosome mosaicism and Y-derived-material and genetic findings were correlated to clinical data.

**Results:**

Thirty-one participants were enrolled: 18 (58%) had chromosome mosaicisms (FISH), Y-derived material was found in 2. Yet, SRY primer was found with PCR in only one of them and DYZ3 was not found. The most frequent clinical findings were short or webbed neck (81,82%), high-arched palate (78%), breast hypertelorism, e cubitus valgus and genu valgus (57.6%, both), short fourth metacarpals (46.9%), epicanthic folds (43.8%), shield chest (43.8%), lymphedema (37.5%), and low set ears (34.4%). Both patients with Y-derived-material had primary amenorrhea, dyslipidemia and reached the height of 150 cm despite not treated with recombinant growth hormone (GHr). One of them showed 26% of leukocytes with Y-derived material and few clinical findings.

**Conclusions:**

FISH techniques proved efficient in detecting chromosome mosaicisms and Y-derived material and searching in different tissues such as mouth cells is critical due to the possibility of tissue-specific mosaicism. Phenotypical variance in TS may be a signal of chromosome mosaicisms, especially with the presence of Y-derived material.

## INTRODUCTION

Full monosomy of X chromosome or structural abnormalities of one of the sex chromosomes characterize Turner syndrome (TS). Both conditions may present with mosaicisms, with two or more different cell lineages.

TS is usually diagnosed with karyotype using GTG banding. About 50%-60% of the cases present with 45,X monosomy, 5%-10% show structural abnormalities and 30%-40% have mosaic cell lineages, including cells with structurally abnormal X and presence of Y chromosome (5%-10% of mosaic cases) (1-5).

Use of molecular and cytomolecular techniques such as PCR (polymerase chain reaction) and FISH (fluorescence in situ hybridization) has been shown to perform better at detecting chromosome mosaicisms. Therefore, mosaicisms have proven to be more frequent than monosomy (1,6). Detecting Y-derived-material in TS is of great relevance for defining prognosis, for its presence increases risk of gonadal tumors, specially gonadoblastoma, as well as virilization and hyperandrogenism (7,8).

The objective of this work was to investigate the frequency of chromosome mosaicisms, especially with presence of Y-derived-material, in patients with 45,X TS with PCR and FISH techniques performed in mouth epithelium samples.

## MATERIALS AND METHODS

ST patients followed at Genetics Clinics of University Hospital (Edgar Santos Hospital – Federal University of Bahia) were screened for the study between May 2014 and December 2015.

Participants were considered eligible if they had 45,X karyotypes (GTG banding). Research Ethics Committee of the hospital approved the study protocol (CAAE: 36305314.5.0000.0049). All 31 patients were evaluated by the multidisciplinary team (pediatricians, endocrinologists and geneticists) and clinical data and biological samples were collected.

### Laboratory methods

#### FISH in mouth epithelial cells

Mouth epithelial cells (buccal smears) were collected from the inner lining of the cheeks after mouth-washing with saline solution. The sample was centrifugated for 15 minutes at 1,000 rpm, incubated at KCl solution at 37 °C for 10 minutes, followed by prefixation with Carnoy solution (3:1 methanol/acetic acid) (9). Afterwards, the slide was washed out in different solutions: 2xSSC for 1 h at 37 °C; pepsin 0.005% (Pepsin/H_2_O/HCl 1%) for 13 minutes at 37 °C; PBS at room temperature (RT) for 5 minutes; 0.95% formaldehyde for 5 minutes at RT; PBS solution for 5 minutes at RT; alcoholic series (70%, 85% and 100%, respectively) at RT for 2 minutes in accordance with Cytocell probe protocol.

For hybridization, two alpha satellite probes were used: DXZ1 for X chromosome and DYZ3 for Y chromosome (Cytocell-UK), green and orange spectra, respectively.

About 200 nuclei for each participant were analyzed in fluorescence microscopes with appropriate filters.

#### PCR in buccal smears

DNA extraction was performed with the following techniques: samples were centrifugated for 15 minutes at 1,000 rpm; 10% Chelex was added; double boiling incubation for 10 minutes, cooling with ice; centrifugation for 30 seconds; supernatant was transferred to another pre-identified microtube and freezer stored at -20 °C. Primers for SRY (242 bp) and DYZ3 (1,000 bp) were obtained from Araújo’s (10) sequencing and ring formation temperatures, according to Ventura’s protocol (11) adopted by the authors. For reaction controls, a SNRP gene (located at 15q11.2) primer (145 bp) was used. Banding patterns were analyzed Amplified DNA fragments were separated on a 1% agarose gel for DYZ3 + SNRPN and a 3% agarose gel for SRY + SNRPN. The gel was stained with 12 μL of Sybr Safe per 100 mL. banding patterns were analyzed by comparing their measurements with the markers of molecular weight. One positive and one negative controls were used for each amplification.

## RESULTS

Table 1 shows FISH results. 13 out of 41 (42%) participants showed only one signal of hybridization of X centromere, corresponding to X monosomy. Eighteen showed two signals of X or Y chromosomes, representing mosaicisms. X/XX mosaicisms were seen in 13 participants, X/XX/XXX in 2 and Y-derived chromosome mosaicism in 3 participants.

**Table 1:** FISH analysis in mouth epithelial cells

Patient	FISH	N of studied nuclei	X %	XX %	XXX %	XY %	XYY %
**TS01**	nuc ish(DXZ1)x1[200]	200	100				
**TS02**	nuc ish(DXZ1)x1[191]/(DXZ1)x2[11]	202	94	6			
**TS03**	nucish(DXZ1)x1[64]/(DXZ1)x2[135]/ (DXZ1)x3[12]	211	30	64	6		
**TS04**	nuc ish(DXZ1)x1[88]/(DXZ1)x2[12]	100	88	12			
**TS05**	nucish(DXZ1)x1[148]/(DXZ1,DYZ3)x1[29]/ (DXZ1x1,DYZ3x2)[23]	200	74	-	-	14.5	11.5
**TS06**	nuc ish(DXZ1)x1[198]/(DXZ1)x2[2]	503	98.6	1.4			
**TS07**	nuc ish(DXZ1)x1[195]/(DXZ1)x2[5]	200	97,5	2.5			
**TS08**	nuc ish(DXZ1)x1[192]/(DXZ1)x2[8]	200	96	4			
**TS09**	nuc ish(DXZ1)x1[196]/(DXZ1)x2[4]	200	98	2			
**TS10**	nuc ish(DXZ1)x1[200]	200	100				
**TS11**	nuc ish(DXZ1)x1[196]/(DXZ1)x2[4]	200	98	2			
**TS12**	nuc ish(DXZ1)x1[196]/(DXZ1,DYZ3)x1[8]	204	96	-	-	4	
**TS13**	nuc ish(DXZ1)x1[136]/(DXZ1)x2[64]	200	68	32			
**TS14**	nuc ish(DXZ1)x1[200]	200	100				
**TS15**	nuc ish(DXZ1)x1[190]	190	100				
**TS16**	nuc ish(DXZ1)x1[200]	200	100				
**TS17**	nuc ish(DXZ1)x1[200	200	100				
**TS18**	nuc ish(DXZ1)x1[200]	200	100				
**TS19**	nuc ish(DXZ1)x1[167]/(DXZ1)x2[33]	200	83.5	16.5			
**TS20**	nuc ish(DXZ1)x1[506]/(DXZ1,DYZ3)x1[1]	507	99.9	-	-	0.1	
**TS21**	nuc ish(DXZ1)x1[167]/(DXZ1)x2[35]	202	82.7	17.3			
**TS22**	nuc ish (DXZ1)x1[500]/(DXZ1)x2[3]	503	99.5	0.5			
**TS23**	nuc ish(DXZ1)x1[155]/(DXZ1)x2[45]	200	78	22			
**TS24**	nuc ish(DXZ1)x1[200]	200	100				
**TS25**	nuc ish(DXZ1)x1[196]/(DXZ1)x2[4]	200	98	2			
**TS26**	nuc ish(DXZ1)x1[200]	200	100				
**TS27**	nuc ish (DXZ1)x1[111]/(DXZ1)x2[88]/(DXZ1)x3[1]	200	55.5	44	0.5		
**TS28**	nuc ish(DXZ1)x1[200]	200	100				
**TS29**	nuc ish(DXZ1)x1[200]	200	100				
**TS30**	nuc ish(DXZ1)x1[200]	200	100				
**TS31**	nuc ish(DXZ1)x1[200]	200	100				

Eight out of eighteen participants had over 10% chromosome mosaicism, seven had 2%-10% mosaicism and three under 2% (even after expanding analysis to 500 nuclei). Among the participants with mosaicism over 10%, ST03 was the only one with XX lineage larger than the monosomic lineage.

**Figure 1:**
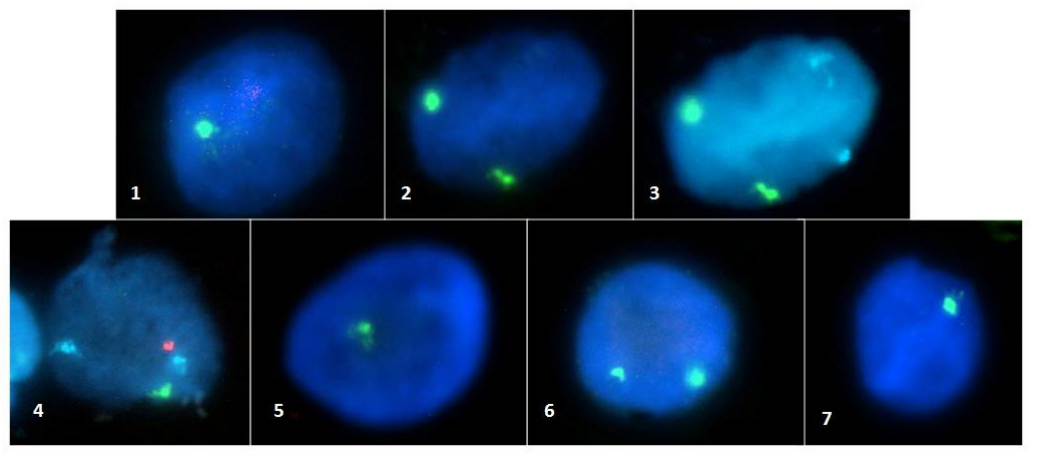
Photographs of interphasic nuclei of TS06 (1, 2 and 3), TS20 (4 and 5) and TS22 (6 and 7). Acqua-colored signals in 3 and 4 correspond to control-probe in centromeric region in chromosome 18, captured in these photographs as a parameter of hybridization of the nuclei.

Figure 1 shows FISH images of participants with mosaicisms under 2%. In slides 3 and 4, one 18-chromosome control probe shows (blue signals) chromosome 18 with double hybridization signals.

**Figure 2:**
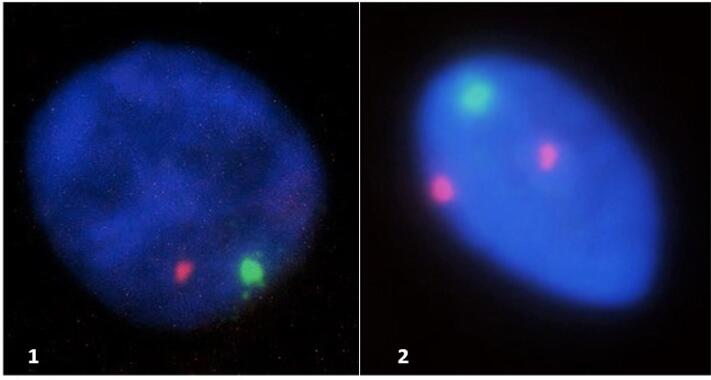
Interphasic nuclei with Y-chromosome. TS12 (1) and TS05 (2).

ST05, one of the participants with chromosome Y, showed two lineages with Y derived material, which adds up to 26% of the cells. ST12 had 4% Y-derived material whereas ST20 showed only one and needs further studies to confirm mosaicism (Figure 2).

**Figure 3:**
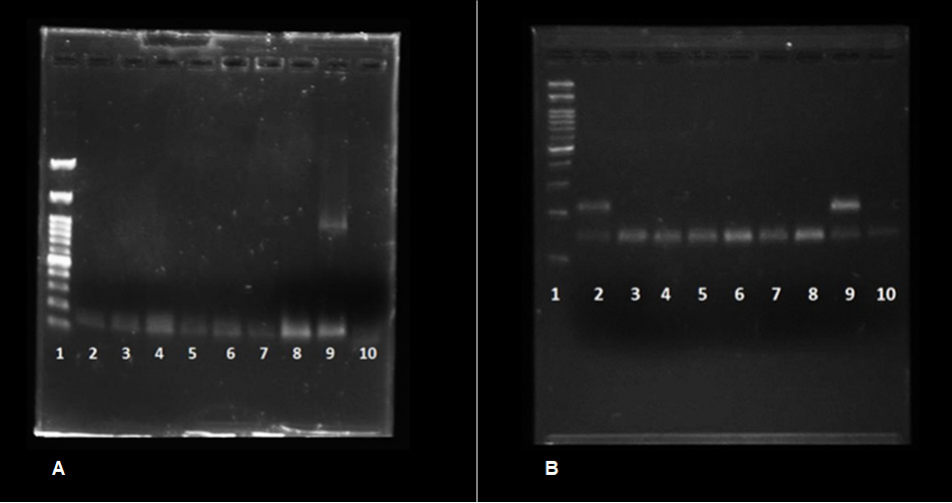
PCR Results: **A**) Primer DYZ3 + SNRPN. Bands: 1. Marker of molecular weight; 2. TS05; 3. TS10; 4. TS12; 5. TS14; 6. ST20; 7. TS22; 8. TS32; 9. Positive Control; 10. White; **B**) Primer SRY + SNRPN. Bands: 1. Marker of molecular weight; 2. TS05; 3. TS10; 4. TS12; 5. TS14; 6. TS20; 7. TS22; 8. TS32; 9. Positive Control; 10. Negative Control.

DYZ3-PCR studies did not show presence of this region in any of the studied participants, whereas SRY-PCR was present only in ST05 (Figure 3).

The participants of the present study had mean age of 10.91 ± 8.9 years, ranging 1 month to 35 years. Eighteen of them had been referred due to dysmorphisms and/or short stature and the others (45.5%) due to primary amenorrhea at pubertal age and might not have other physical alterations.

Participants showed variable number of dysmorphic features, range 0-17, average 8.3 (SD 4.4). The most frequent features were: short or webbed neck (81.8%), followed by high-arched palate (78%), breast hypertelorism and cubitus and genu valgum (57.6%), short fourth metacarpal (46.9%), epicanthic folds (43.8%), shield chests (43.8%), lymphoedema (37.5%), low-set ears (34.4%) (Table 2).

**Table 2:** TS dysmorphic features found in participants, grouped by FISH results

Features	Monosomy (N = 13)	Mosaicism > 10% (N = 7)	Mosaicism < 10% and ≥ 2% (N = 6)	Mosaicism < 2% (N = 2)	X-Marker Mosaicism (N = 2)	Y-derived Marker Mosaicism (N = 2)	Total
Short or webbed neck	13	4	5	2	1	1	26
High-arched palate	12	5	5	2	NE	1	25
Breast hypertelorism	8	5	4	2	NE	0	19
Cubitus valgus and e genu valgum	9	5	2	1	2	0	19
Low posterior hairline	10	2	4	1	1	0	18
Shortened 4th metacarpal	8	4	3	0	0	0	15
Epicanthic folds	7	1	4	1	1	0	14
Shield chest	6	1	4	1	2	0	14
Lymphedema in early life	7	1	3	1	0	0	12
Low-set ears	4	3	2	1	1	0	11
Deformity of external ear	7	0	2	2	0	0	11
Nail dysplasia	6	1	1	0	0	0	8
Bushy brows	4	0	2	0	0	0	6
Abnormal dental development	3	1	1	1	0	0	6
Eye hypertelorism	3	0	1	0	0	1	5
Pectus excavatum	2	1	0	1	0	0	4
Ptosis	0	0	2	1	0	0	3
Seborrheic dermatitis	2	1	0	0	0	0	3
*Multiple pigmented nevi*	0	2	0	0	0	0	2
Micrognathia	0	0	1	0	0	0	1
Kyphosis	0	0	0	1	0	0	1

NE: patient not evaluated for the condition.

The most frequent comorbidities found in 17 (53.1%) the participants were endocrine conditions (low bone mass and hypothyroidism) followed by cardiac malformations (24.2%, especially aortic coarctation) and renal malformations (15.1%), eye abnormalities (3 cases), liver hemangioma (1 case) and hepatic steatosis (1 case).

## DISCUSSION

Many studies have reported usefulness of PCR and FISH techniques to detect cryptic mosaicism, especially in non-blood cells, buccal smear cells in particular. Nazarenko and cols. (12) and Freriks and cols. (9) evaluated 45,X patients’ buccal smear cells with FISH and found mosaicisms in 29% and 30.2% of them, respectively. In the present study, mosaicism was found in 58% of the participants. In two of them (6.4%), Y-derived chromosome was found, excluded one case presenting Y-mosaicism (TS20) in only one cell.

Our results suggest further investigation other than peripherical G-banding karyotyping may be necessary in 45,X patients since Y-derived material may be associated with elevated risk of gonadoblastoma in women with TS.

In the present study, Y-chromosome was detected in only 3.2% of the participants. In similar studies, different prevalences were found: Bianco and cols. (2) found SRY in 35% and DYZ3 in 10% of the participants, respectively; and DYZ3 amplification was found only combined with SRY amplification. In 2009, the same author (13) found SRY in 12 patients and only four were also positive for DYZ3. Ventura (11) found SRY in 3% out of 98 patients in several tissues; 3 other primers were used, and SRY was amplified in 3 out of four cases with Y-derived material. Cortés-Gutiérrez and cols. (14) found SRY in 1 out of 24 patients (4,2%); Freriks and cols. (9) found SRY in none of five patients with Y chromosome detected with FISH in mouth epithelial cells; yet, 2 of them were positive using RT-PCR.

These data show that, among the most popular primers, SRY is the most frequently detected in women with TS. Bianco and Bianco and cols. (2,13) also used SRY and DYZ3 primers, the latest one was found only in combination with SRY but not in the totality of the cases, as our own data also show (DYZ3 found in neither of our 3 cases with Y-derived material detected with FISH, not even in the SRY-positive patient (TS05) who presented 26% of cells with Y chromosome signal by FISH, which may reflect the non-completeness of this chromosome in patients.

Other genes have been used in the investigation of hidden Y chromosome sequences in patients with TS, among them we highlight: ZFY, DYZ1, DYS1, PABY, and TSPY. The last one, TSPY, has great relevance because it seems to be associated with the GBY region – an oncogenic locus found on the Y chromosome – and it showed high expression in some patients with gonadoblastoma. The SRY and DYZ3 genes, in turn, are the sequences most commonly used in studies, and play a role in sexual determination and chromosomal stability, respectively (8,15).

Similar to Freriks’s and cols. (9) findings, the present study showed that FISH was more efficient than PCR when investigating Y-chromosome mosaicism because FISH makes it possible to characterize the chromosomes of the studied patients and rule out cases presenting Y-derived chromosomes and detect a second lineage with two or more X-derived chromosomes.

This study also showed that using mouth epithelial cells may be efficient for this kind of analysis and advantageously evaluates a tissue with the same embryo origin as fibroblasts without the need for biopsy or culturing. Yet, it requires same-day processing to avoid bacterial contamination (16).

Several studies have evaluated the usefulness of analyzing tissues with different embryo origin than lymphocytes, whose cultures are generally used for karyotyping. In the present study, 7 (38%) out of the 18 patients presenting mosaicisms, at least one additional X was found in over 10% of the studied cells. Three of them showed elevated rates of a second chromosomic lineage (64%, 44% e 32%). Such data highly suggest the presence of tissue-specific mosaicisms, since those lineages had not been found in lymphocyte karyotyping, even being detected in elevated rates.

Several studies have shown tissue-specific differences in detecting aneuploidy in women with TS. Nazarenko and cols. (12) found tissue-specific differences in 92% of the studied cases; Guedes (3) described a case whose lymphocyte karyotype showed 2.5% of the cells to be 45,X and 97,5% idic(Yp) but 60% of her gonadal cells presented 45,X lineage. Hanson and cols. (6) studied mosaicisms with FISH technique in lymphocytes and mouth cells in 45,X patients found four cases with high-frequency 46,XX mosaicisms and one of them with 60% 47,XXX mosaicism in mouth cells.

Previous studies with Brazilian patients showed similar mean age at diagnosis compared to this series (10.9 years) (4,17,18).

Patients were referred during adolescence for several reasons: primary amenorrhea and lack of pubertal signs were the most frequent. During childhood (ages 0-5 years), they were all (100%) referred due to dysmorphisms or congenital malformations. Yet, dysmorphisms were more frequently found in girls referred at ages 6-11 years.

Among the girls diagnosed after the age of 12 years (n = 17), at least 7(41%) showed at least seven dysmorphisms due to TS: short or webbed neck, high-arched palate, valgus cubitus and genu valgum, shortened fourth metacarpal, breast hypertelorism, low set hair and epicanthus. Considering the relevance of early diagnosis of TS to enhance life quality, diagnosis could have been made earlier since dysmorphisms were present since birth.

Considering the clinical profile of the patients, we observed that the percentages of each characteristic are very varied among the works found in the literature, with the same characteristic presenting high frequency (>50%) in some studies and low frequency in others. This can be seen in the study of Jung and cols. (4) which refers to the presence of ulna valgus in 72.5% of the cases and ogival palate in 59.6%, and the work by Araújo and cols. (19) who reported 22.4% ulna valgus and 16.6% ogival palate. In the current work, the values for ulna valgus were 57.9% and 78% for ogival palate, contributing to the perception of the great variability observed in the literature, which may be associated with factors such as underdiagnosis and a greater tendency to refer patients with more dysmorphisms. Despite this wide variation, the majority of studies report the presence of the same characteristics in the population studied with TS (20-23).

The most frequent conditions associated with TS are metabolic and endocrine dysfunction, heart and kidney diseases, ear infections, liver abnormalities and ophthalmological conditions. Frequencies of such conditions also vary in literature, yet less often than dysmorphisms. The present study found endocrine and/or metabolic conditions in 51.1% of the cases, whilst Guimarães and cols. (20) and Araújo and cols. (19) found them in 38.3% and 21% of their cases, respectively.

Eight (24.2%) participants had heart malformations, such as described by Guimarães and cols. (20) (25%). Araújo and cols. (19) found heart disease in 45% of their TS cases.

TS03, who presented with additional X-lineage in 70% of the studied cells, was diagnosed at 25 years of age due to secondary amenorrhea. She has short stature (138 cm), epicanthus, high-arched palate, cubitus valgus, genu valgum, shortened fourth metacarpus and low bone mass. She also had a medial tumor in conjunctiva of her right eye. The large proportion of 46,XX lineage could explain menarche and breast development and delayed diagnosis.

The two participants with Y chromosomes (TS05 e TS12) both had primary amenorrhea, dyslipidemia and were 150 cm tall despite not being treated with GH. In the present study, only one other patient, with mosaicism of X > 10%, presented dyslipidemia and 6 (19.3%) patients had primary amenorrhea, of which 1 presented monosomy X, 3 mosaicism of X < 2%, and 2 mosaicism of X > 10%. No other patients reached the final stature of 1.5 m, even with the use of GH. Of the patients who used the hormone, the highest stature was 1.45 m, and in the other patients who did not use the hormone, the highest stature was also 1.45 m.TS05 had very few dysmorphic features: eye hypertelorism, downward lips, high-arched palate, short neck and low bone mass. TS12 had only 4% Y-derived cells and had no dysmorphisms but had hypothyroidism, liver hemangioma and panic syndrome. Gonadal study has not been performed yet in these patients.

TS20 showed Y-derived material in one out of the 507 studied nuclei and was diagnosed at the age of 15 years due to failure to thrive, short stature and pubertal delay. She was on GHr for only 18 months and final height was 136 cm at the age of 21 years. She has hypogonadism, low bone mass and mild hearing deficit. Her only dysmorphism is short neck in addition to short stature.

In the present study, FISH technique in mouth cells proved efficient to detect chromosomal mosaicisms in patients with TS and karyotype 45,X and FISH can be added to traditional G-banding techniques in lymphocytes to search chromosomal mosaicisms and Y-derived lineages. If high-frequency chromosomal mosaicisms are found, studying a different tissue may be relevant due to the possibility of tissue-specific mosaicism.

FISH was shown more efficient than PCR at identifying chromosomal mosaicisms with Y-derived material due to the fact that PCR is more specific than FISH. However, PCR can be used to investigate chromosomal mosaicism in women with TS as a screening for Y-specific sequences. This may be more cost-effective, as in the current study, it was observed that the costs of the reagents for carrying out the FISH were three times higher than for PCR, as well as which the FISH equipment is more expensive.

Although there is a great phenotypical variability among patients with TS, clinical features are well established, being short stature the most prevalent and it should be used as a marker to suspect TS.

In the present study, age at diagnosis was inversely correlated to dysmorphisms: the richer the phenotype, the earlier they were referred to specialized care. Those diagnosed age 12 years and over were referred to the clinic for amenorrhea.

Establishing precisely the chromosomal abnormalities is extremely important not only to diagnose TS but for prognostic reasons since the presence of lineages with additional X chromosomes or the presence of Y chromosome influence phenotype and risk of malignancy.
